# PARM-1 Is an Endoplasmic Reticulum Molecule Involved in Endoplasmic Reticulum Stress-Induced Apoptosis in Rat Cardiac Myocytes

**DOI:** 10.1371/journal.pone.0009746

**Published:** 2010-03-18

**Authors:** Koji Isodono, Tomosaburo Takahashi, Hiroko Imoto, Naohiko Nakanishi, Takehiro Ogata, Satoshi Asada, Atsuo Adachi, Tomomi Ueyama, Hidemasa Oh, Hiroaki Matsubara

**Affiliations:** 1 Department of Cardiovascular Medicine, Kyoto Prefectural University of Medicine, Kyoto, Japan; 2 Department of Experimental Therapeutics, Translational Research Center, Kyoto University Hospital, Kyoto, Japan; University of Cincinnati, United States of America

## Abstract

To identify novel transmembrane and secretory molecules expressed in cardiac myocytes, signal sequence trap screening was performed in rat neonatal cardiac myocytes. One of the molecules identified was a transmembrane protein, prostatic androgen repressed message-1 (PARM-1). While PARM-1 has been identified as a gene induced in prostate in response to castration, its function is largely unknown. Our expression analysis revealed that PARM-1 was specifically expressed in hearts and skeletal muscles, and in the heart, cardiac myocytes, but not non-myocytes expressed PARM-1. Immunofluorescent staining showed that PARM-1 was predominantly localized in endoplasmic reticulum (ER). In Dahl salt-sensitive rats, high-salt diet resulted in hypertension, cardiac hypertrophy and subsequent heart failure, and significantly stimulated PARM-1 expression in the hearts, with a concomitant increase in ER stress markers such as GRP78 and CHOP. In cultured cardiac myocytes, PARM-1 expression was stimulated by proinflammatory cytokines, but not by hypertrophic stimuli. A marked increase in PARM-1 expression was observed in response to ER stress inducers such as thapsigargin and tunicamycin, which also induced apoptotic cell death. Silencing PARM-1 expression by siRNAs enhanced apoptotic response in cardiac myocytes to ER stresses. PARM-1 silencing also repressed expression of PERK and ATF6, and augmented expression of CHOP without affecting IRE-1 expression and JNK and Caspase-12 activation. Thus, PARM-1 expression is induced by ER stress, which plays a protective role in cardiac myocytes through regulating PERK, ATF6 and CHOP expression. These results suggested that PARM-1 is a novel ER transmembrane molecule involved in cardiac remodeling in hypertensive heart disease.

## Introduction

Chronic heart failure is a major and increasing public health problem, especially in industrialized societies with aging populations. The rate of hospital admission has increased progressively over the past years, making heart failure one of the most common indications for hospital admission in elderly people [1]. Considerable therapeutic advances including pharmacotherapy such as blockade of renin-angiotensin system and β adrenergic receptor, and nonpharmacologic therapies such as heart transplantation and resynchronization therapy have been made in recent years. However, mortality among patients with heart failure remains still substantial, and the well-beings deteriorate dramatically, underscoring the need for additional therapeutic options [2]. Since there may be significant potential in therapies targeting the novel pathological pathways, it is crucial to understand the molecular mechanisms involved in cardiac pathophysiology, especially ones specifically operated in the hearts.

Apoptosis is a process of innate cellular death, controlled by complex and diverse molecular mechanisms with considerable cell type specificity. Apoptosis plays important roles in various aspects of biology from development to a wide range of diseases such as cancers and cardiovascular diseases. In the heart, apoptosis is essential for cardiac development such as formation of cardiac valves and outflow tract [3]. Although apoptosis is rare in normal human hearts, the rate of cardiac myocyte apoptosis can increase several hundred fold in dilated and ischemic cardiomyopathies, hypertensive heart disease and arrhythmogenic right ventricular dysplasia, and an association between apoptosis, cardiac myocyte loss, ventricular remodeling and deterioration of systolic performance has been demonstrated in multiple experimental models [4], [5]. Although apoptotic processes are tightly regulated by extracellular factors and intracellular signalings, the precise molecular mechanisms governing cardiac myocyte apoptosis have not been fully elucidated, and understanding the regulation of apoptosis is of great importance for the advancement of cardiac biology and for developing novel therapeutic strategies.

In this study, we sought to identify a novel molecule involved in cardiac pathophysiology using efficient signal sequence trap method. Signal sequence trap is a strategy to specifically clone cDNA fragments with signal sequence, a short hydrophobic stretch of amino acids which mediates targeting of secreted and cell-surface proteins to the cell membrane [6], [7]. As secreted and membrane molecules play critical roles in cellular functions and interactions, and are potential therapeutic targets for antagonistic or agonistic strategies, this strategy could be useful to identify novel molecules involved in cardiac pathophysiology. Among the molecules identified, in this study, we analyzed the role of prostatic androgen repressed message-1 (PARM-1) [8] in cardiac myocytes.

## Results

### Identification of PARM-1 as an endoplasmic reticulum protein expressed in cardiac myocytes

**Figure 1:**
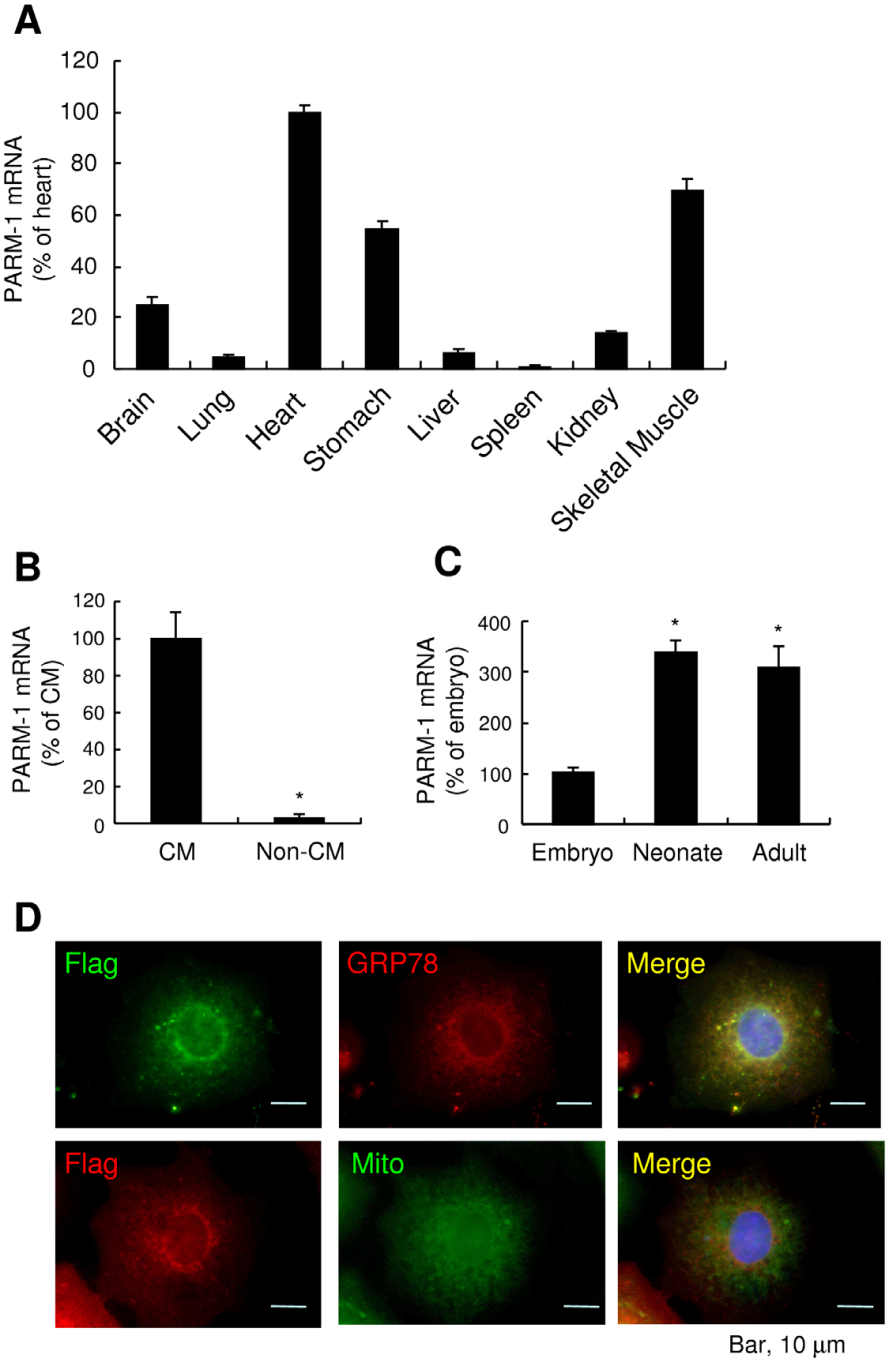
PARM-1 is an ER protein expressed in cardiac myocytes. *A, B, C*: PARM-1 expression was assessed by kinetic real-time RT-PCR in various tissues of adult mice (A), cultured rat neonatal cardiac myocytes and non-myocytes (B), and developmental mouse hearts (C), respectively. *P<0.05 versus cardiac myocytes (B) or embryonic hearts (C). *D*: Cellular localization of PARM-1 was analyzed by immunostaining of cultured neonatal rat cardiac myocytes expressing flag-tagged PARM-1 with anti-Flag antibody (left), and anti-GRP78 antibody or MitoTacker (center). Nuclei were stained by DAPI.

In this study, we applied the efficient signal sequence trap cloning using retrovirus-mediated gene transfer to identify novel transmembrane and secreted molecules expressed in cardiac myocytes [6], [9]. Among the molecules identified (Table 1), a transmembrane protein, PARM-1, was selected for further analysis, because its expression and functions in cardiac myocytes were largely unknown. Our expression analysis revealed that PARM-1 was most abundantly expressed in hearts (Fig. 1A). PARM-1 was also expressed in skeletal muscles and stomachs. These results suggested that PARM-1 could be expressed in striated and smooth muscles. To identify a cell type expressing PARM-1 in the hearts, we separated cardiac myocytes and non-myocytes from neonatal hearts, and expression of PARM-1 in cardiac myocytes and non-myocytes was analyzed. As shown in Fig. 1B, PARM-1 transcript was specifically expressed in cardiac myocytes, but not in non-myocytes, indicating that the major source of PARM-1 in the hearts is cardiac myocytes. To evaluate how PARM-1 expression is regulated during heart development, we analyzed mRNA expression in hearts of embryos, neonates and adult mice (Fig. 1C). PARM-1 mRNA expression in the heart was detected at embryonic day 10.5 (E10.5), increased until neonatal stages and, thereafter remained unchanged through adult stages. To assess subcellular localization of PARM-1 in cardiac myocytes, flag-tagged PARM-1 was expressed in cultured neonatal cardiac myocytes, and the cells were stained with anti-flag antibody. PARM-1 staining showed cytoplasmic vesicular network pattern with intense perinuclear localization (Fig. 1D). When the cells were co-stained with anti-78 kDa glucose regulated protein/BiP (GRP78) antibody or MitoTracker, PARM-1 was co-localized with GRP78, an endoplasmic reticulum (ER)-resident chaperone, but not with MitoTracker, a mitochondrion-selective dye (Fig. 1D). Thus, PARM-1 is a transmembrane protein predominantly localized in ER in cardiac myocytes.

**Table 1:** Signal sequence trap screening of cultured neonatal cardiac myocytes.

Symbol	protein name	clone number
Spark	secreted acidic cysteine rich glycoprotein	33
Nppa	natriuretic peptide precursor type A	13
Tmem9	transmembrane protein 9 (predicted)	10
Coll8a1	procollagen, type XVIII, alpha 1	10
Podxl	podocalyxin-like	8
Gpc1	glypican 1	6
Fxyd5	FXYD domain-containing ion transport regulator 5	5
Parm1	prostatic androgen repressed message 1	5
Col4a1	procollagen, type IV, alpha 2 (predicted)	4
Col1a1	procollagen, type 1, alpha 1	4
Srl	sarcalumenin	4
Sdc3	syndecan 3	4
Jun	Jun oncogene	3
Dlk1	delta-like 1 homolog (Drosophila)	3
Igfbp3	insulin-like growth factor binding protein 3	3
Col4a2	procollagen, type IV, alpha 1	2
Pi16	protease inhibitor 16 (predicted)	2
Notch3	Notch gene homolog 3	2
Tmem9 sf4	transmembrane 9 superfamily protein member 4	1
RGD1562476	similar to Eso3 protein (predicted)	1
Pecam	platelet/endothelial cell adhesion molecule	1
Gnai2	guanine nucleotide binding protein, alpha inhibiting 2	1
Sparcl1	SPARC-like 1	1
Atp6ap2	ATPase, H+ transporting, lysosomal accessory protein 2	1
Myadm	myeloid-associated differentiation marker	1
Apoe	apolipoprotein E	1
Fstl1	follistatin-like 1	1
Hspa5	heat shock 70 kDa protein 5 (glucose-regulated protein)	1
Sfrp1	secreted frizzled-related protein 1	1
Cd320	CD320 antigen	1
Canx	Calnexin	1

### PARM-1 expression is increased in hearts of hypertensive heart disease

**Figure 2:**
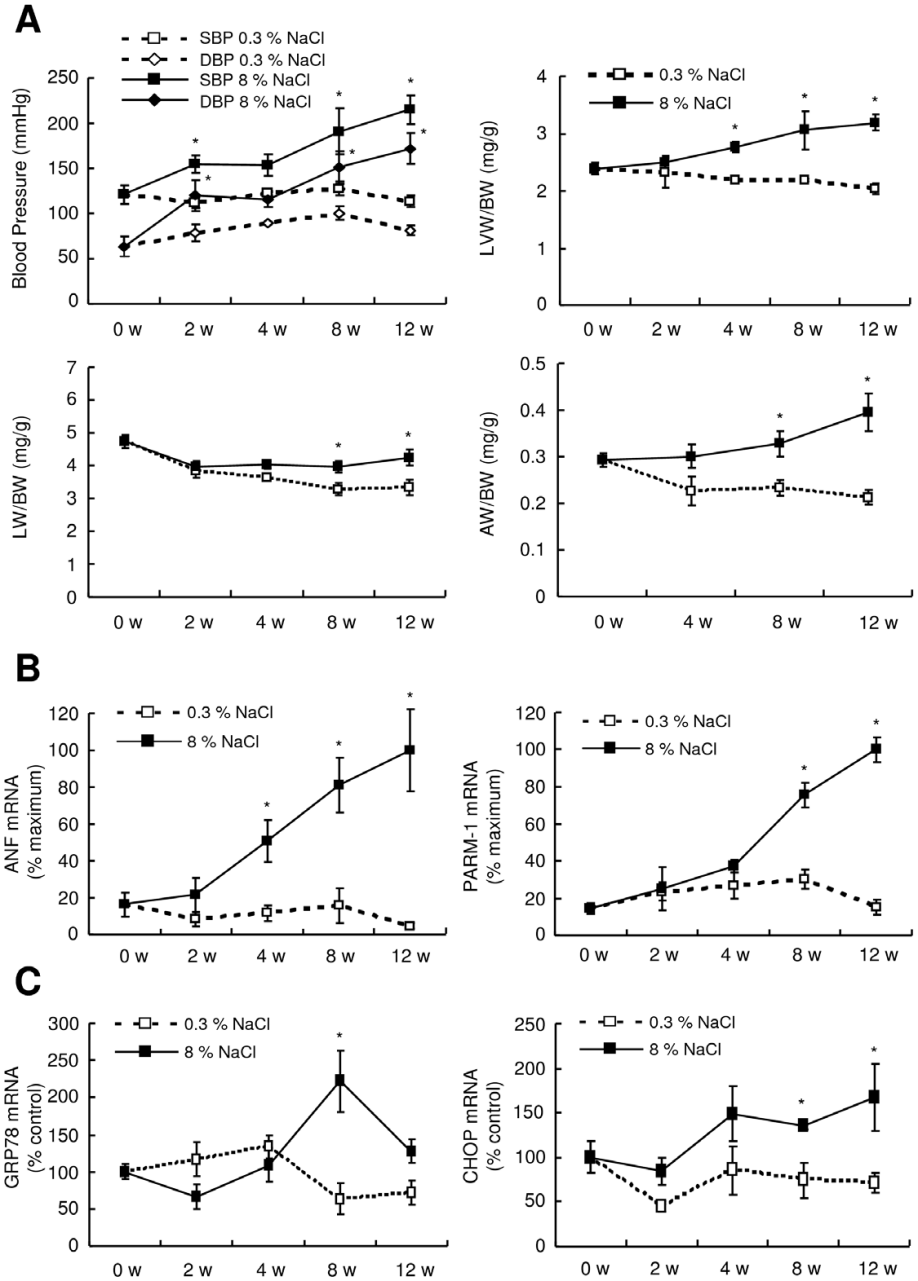
PARM-1 expression and ER stress response was activated in hypertensive heart disease model of Dahl salt-sensitive rats. *A*: The animals were subjected to a high- (8% NaCl, n = 12 for each time point) or low-salt diet (0.3% NaCl, n = 8 for each time point). Blood pressure and left ventricular weight (LVW), Body weight (BW), lung weight (LW) and atrial weight (AW) were measured at the indicated periods of time after starting the designated diet. *B, C*: ANF and PARM-1 expression (B), and ER stress markers such as GRP78 and CHOP (C) were analyzed by kinetic real-time PCR on cDNAs from the hearts of Dahl salt-sensitive rats. *P<0.05 versus a low-salt diet group at the respective time point.

To investigate whether PARM-1 expression was regulated under pathological conditions in the postnatal hearts, we analyzed PARM-1 expression in the hearts of Dahl salt-sensitive rats. Dahl salt-sensitive rats were randomly assigned to receive either a 0.3% NaCl (low-salt) diet or an 8% NaCl (high-salt) diet at the age of 6 weeks. Consistent with the previous reports [10], [11], upon a high-salt diet, Dahl salt-sensitive rats developed systemic hypertension, and a subsequent increase in left ventricular weight (LVW) to body weight (BW) ratio until 4 weeks after starting diet (Fig. 2A), indicating the development of left ventricular hypertrophy. Thereafter, a significant increase in lung weight (LW) to BW ratio and atrial weight (AW) to BW ratio was observed at 8 weeks (Fig. 2A), which was concomitant with a marked increase in atrial natriuretic factor (ANF) expression (Fig. 2B), suggesting the transition from hypertrophy to heart failure. In this model, PARM-1 expression was significantly increased at 8 weeks after starting diet, and reached more than 5 fold increase at 12 weeks (Fig. 2B). As ER stress is recently implicated in the pathogenesis of heart diseases such as ischemic heart disease and heart failure [12], [13], we analyzed the expression of ER stress markers such as GRP78 and C/EBP homologous protein transcription factor (CHOP) in this model, and found that these ER stress markers were induced at the phase of transition from hypertrophy to heart failure (Fig. 2C).

### Inflammatory cytokines and ER stress augment PARM-1 expression in cardiac myocytes

**Figure 3:**
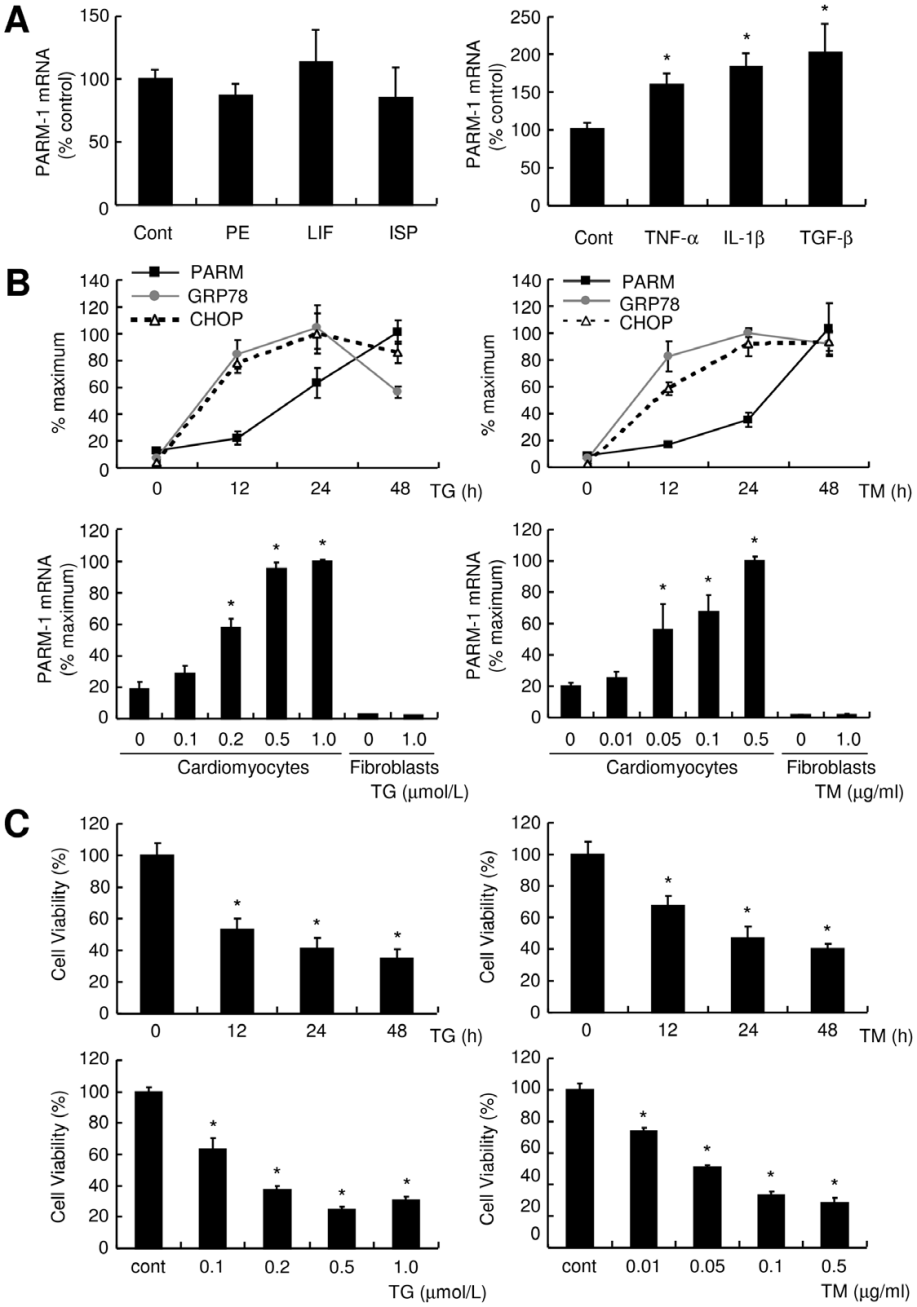
PARM-1 expression was induced by inflammatory cytokines and ER stress inducers specifically in cardiac myocytes. *A*: Cultured rat neonatal cardiac myocytes were stimulated by hypertrophic stimuli such as 100 µmol/l phenylephrine (PE), 1000 U/ml leukemia inhibitory factor (LIF) and 10 µmol/l isoproterenol (ISP), or inflammatory cytokines such as 100 ng/ml TNF-α, 5 ng/ml IL-1β and 4 ng/ml TGF-β. PARM-1 expression was analyzed 48 hours after stimulation. *B*: Cardiac myocytes were treated with 0.5 µmol/l thapsigargin (TG) or 0.1 µg/ml tunicamycin (TM) for the indicated periods of time, and GRP78, CHOP and PARM-1 expression was analyzed by kinetic real time PCR. *C*: Cardiac myocytes and fibroblasts were treated with TG or TM for 48 hours at the indicated concentration, and PARM-1 expression was analyzed. *D*: Cardiac myocytes were treated with TG or TM as indicated, and cell viability was assessed by WST-8 assay. *P<0.05 versus non-treated control cells.

To explore how PARM-1 expression was regulated, cultured cardiac myocytes were stimulated by various hypertrophic stimuli or cytokines, and PARM-1 expression was analyzed by real time PCR. As shown in Fig. 3A, PARM-1 expression was stimulated by proinflammatory cytokines such as TGF-β, TNF-α and IL-1β, but not by hypertrophic stimuli such as phenylephrine, leukemia inhibitory factor and isoproterenol. Since ER stress developed in hypertensive heart failure (Fig. 2C), we also analyzed PARM-1 expression in ER stress conditions (Fig. 3B). Both thapsigargin and tunicamycin treatments led to induction of GRP78 and CHOP in cardiac myocytes. PARM-1 expression was markedly increased 24 hours after treatment with these ER stress inducers, and continued to be increased up to 48 hours (Fig. 3B). While thapsigargin and tunicamycin increased PARM-1 expression in a dose-dependent manner in cardiac myocytes, neither of them induced PARM-1 expression in cardiac fibroblasts (Fig. 3B), indicating PARM-1 induction by ER stress is also specific for cardiac myocytes. Treatment with thapsigargin and tunicamycin resulted in reduced viability in cardiac myocytes, and the effects were time- and dose-dependent (Fig. 3C).

### Silencing PARM-1 augments apoptotic cell death induced by ER stress

**Figure 4:**
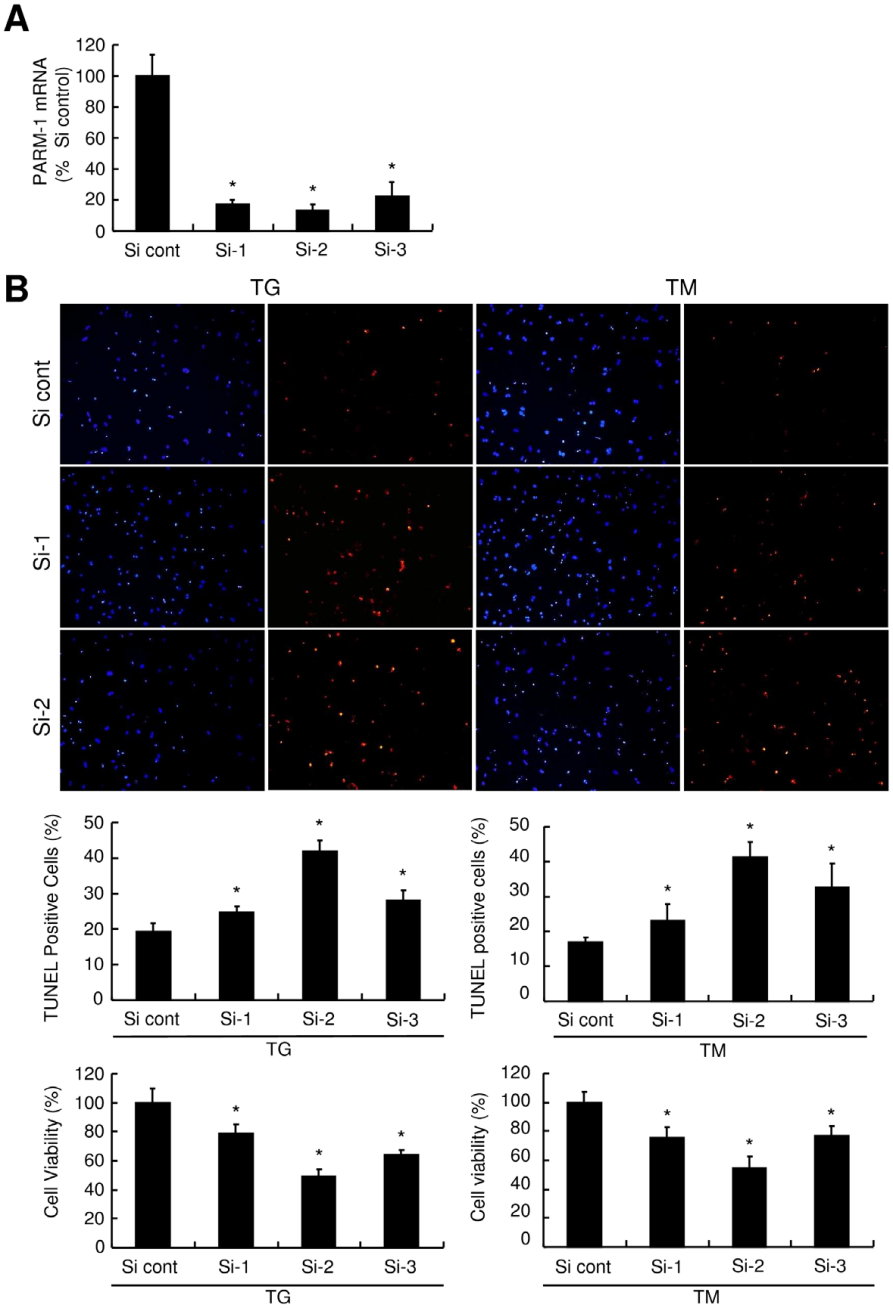
Silencing of PARM-1 augmented apoptotic response to ER stress. *A*: Cultured neonatal rat cardiac myocytes were transfected with 30 nmol/l of three different siRNA duplexes, and assessed for PARM-1 expression 24 hours after transfection. *B*: Cells were treated with TG or TM 72 hours after transfection with siRNAs for 24 hours. Apoptotic cell death was assessed by TUNEL assay, and cell viability was analyzed by WST-8 assay. *P<0.05 versus control siRNA (Si cont).

To assess functional significance of PARM-1 induction in response to ER stress, PARM-1 expression was silenced by siRNA. We identified three different siRNAs, which efficiently silenced PARM-1 expression in cardiac myocytes (Fig. 4A). When PARM-1 expression was silenced by siRNA, ER stress-induced apoptotic response was significantly increased compared to control siRNA (Fig. 4B). Silencing PARM-1 expression further reduced cell viability in response to ER stress inducers. These results indicated that increased expression of PARM-1 in response to ER stress has a protective role in cardiac myocytes.

### PARM-1 silencing decreases PERK and ATF6, and increases CHOP expression in ER stress condition

**Figure 5:**
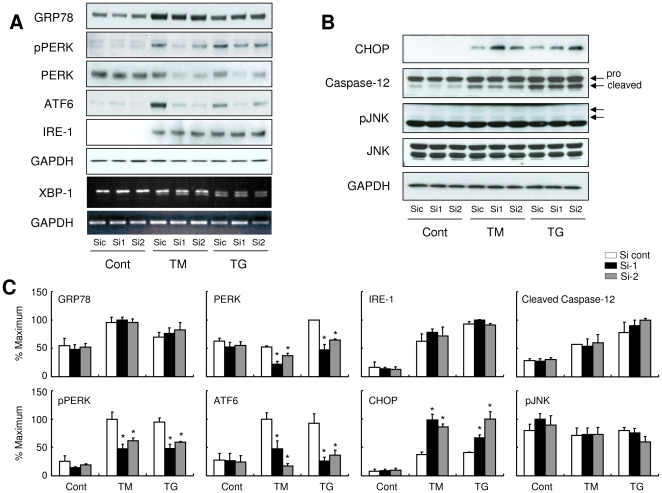
PARM-1 silencing decreases PERK and ATF6, and increases CHOP expression in ER stress condition. Cultured neonatal rat cardiac myocytes were transfected with siRNAs, and, after 72 hours, were treated with TM or TG for 48 hours. *A*: Immunoblot analysis was performed with antibody against GRP78, phospho-PERK, PERK, ATF6, IRE-1 or GAPDH. XBP-1 mRNA splicing was assessed by PCR. *B*: Immunoblot analysis was performed with antibody against CHOP, Caspase-12, phospho-JNK, JNK or GAPDH. *C*: Densitometric analysis was carried out using ImageJ software. The results were normalized against GAPDH, and expressed as percentages of the maximum. *P<0.05 versus control siRNA (Si cont) of respective treatment with TM or TG.

In the last set of experiments, we analyzed the effects of PARM-1 silencing on the signal transduction pathways mediating ER stress responses. In ER stress/unfolded protein response (UPR), misfolded proteins are first recognized by ER-resident chaperons such as GRP78. The status of protein folding in ER lumen is then sensed and transduced by three ER membrane proteins, PKR-like endoplasmic reticulum kinase (PERK), activating transcription factor 6 (ATF6) and endoribonuclease inositol-requiring enzyme-1 (IRE-1), each of which defines a distinct arm of ER stress responses [12], [13], [14]. Expression of GRP78, ATF6 and IRE-1, phosphorylation of PERK and mRNA splicing of XBP-1 were induced by ER stress inducers, thapsigargin or tunicamycin, in cardiac myocytes (Fig. 5A). Although silencing PARM-1 expression did not change GRP78 and IRE-1 expression, and XBP-1 splicing, expression of PERK and ATF6, and phosphorylation of PERK were markedly attenuated by PARM-1 silencing, only in the settings of ER stress (Fig. 5A). It has been shown that ER stress-induced apoptosis signal is mediated by increased expression of CHOP, and activation of Caspase-12 and c-Jun NH2-terminal kinase (JNK) [12], [13], [14]. While silencing of PARM-1 expression did not alter JNK and Caspase-12 activation, CHOP expression by ER stress inducers was significantly enhanced by PARM-1 silencing (Fig. 5B).

## Discussion

In this study, we carried out the efficient signal sequence trap cloning using retrovirus-mediated gene transfer [6], [9] to identify novel transmembrane and secreted molecules expressed in cardiac myocytes. Through this screening, several genes, which have not been widely recognized to be expressed in cardiac myocytes, were identified. These include PARM-1, glypican-1, podocalyxin-like and CD320 antigen, and our expression analysis verified that these genes were indeed expressed in cardiac myocytes (data not shown). As PARM-1 expression was most significantly modulated in the hearts of hypertensive heart disease model of Dahl salt-sensitive rats, we focused PARM-1 in this study. While changes in expression of other genes were not as marked as PARM-1 in this model (data not shown), changes in expression level could not designate the biological significance, and the roles of these other gene products in cardiac myocytes will be an important issue to be studied in the future studies.

PARM-1, also referred to as castration induced prostatic apoptosis-related protein 1 (Cipar1), is originally identified as a gene overexpressed in the prostate of castrated rats [8]. PARM-1 is expressed in the epithelial cells of involuting rat prostates after androgen removal, and the regulation of PARM-1 by androgen is limited to the prostates [8]. In contrast, in prostate cancer cell lines, and in human prostate cancer xenograft, CWR22, PARM-1 is constitutively expressed, and PARM-1 expression is positively regulated by androgen in CWR22 xenograft [15]. While the kinetics of PARM-1 expression is highly correlated with the development of apoptosis after castration, transient expression of PARM-1 does not induce programmed cell death [8]. Although ectopic expression of human PARM-1 in a prostate cancer cell line results in increased colony formation [15], suggesting a probable role of PARM-1 in cell proliferation, the others reported that transient expression of rat PARM-1 does not alter the proliferative property of another prostate cancer cell line [16]. Thus, the regulation of PARM-1 especially in other organs than prostate, and its roles are largely unknown.

Our analysis on hypertensive heart disease model of Dahl salt-sensitive rats demonstrated that PARM-1 expression was significantly upregulated following a high-salt diet. While one of the early cardiac manifestations of this model is hypertension-induced cardiac hypertrophy [10], [11], PARM-1 expression was unchanged 4 weeks after starting a high-salt diet, when a significant increase in LVW to BW ratio was already detected (Fig. 2A,B). Further, in cultured cardiac myocytes, stimulation with hypertrophic stimuli did not alter the expression of PARM-1 (Fig. 3A). This could be in accordance with PARM-1 expression in the hearts during normal developmental stages. While a common feature of various hypertrophic responses is re-activation of fetal gene programs such as ANF and skeletal α-actin genes [17], PARM-1 expression readily detectable at E10.5 was increased toward neonatal stages, and then maintained through adult stages (Fig. 1C). These results suggested that PARM-1 expression could not be a part of fetal gene programs or hypertrophic responses. Another feature of hypertensive heart disease is a development of heart failure. In our model, 8 weeks after staring a high-salt diet, a significant increase in LW to BW ratio was observed, indicating the presence of lung congestion. Although we did not detected a significant increase in LV dimension and a decrease in fractional shortening up to 12 weeks of a high-salt diet (data not shown), a significant increase in AW to BW ratio was noted at 8 weeks, suggesting the development of diastolic heart failure in this model. In this model, PARM-1 was significantly upregulated 8 weeks after starting a high-salt diet, suggesting that PARM-1 was induced in the setting of heart failure. Development of heart failure involves neurohumoral and inflammatory mechanisms [18], [19], and recent evidences have suggested that ER stress plays an important role in the pathogenesis of heart failure [12], [13]. In cultured cardiac myocytes, inflammatory cytokines stimulated PARM-1 expression (Fig. 3A). Further, ER stress inducers, thapsigargin and tunicamycin markedly upregulated PARM-1 expression (Fig. 3B), and ER stress markers such as GRP78 and CHOP were also upregulated in the hearts of hypertensive heart disease model at the heart failure phase (Fig. 2C). These results implied that PARM-1 expression is upregulated during heart failure, involving inflammatory cytokines and ER stress.

As PARM-1 was localized in ER in cardiac myocytes, and ER stress induced PARM-1 expression, PARM-1 could play a role in ER stress response. ER is a cellular organelle, where protein synthesis and folding of secreted and transmembrane proteins take place. When ER environment is perturbed, and the folding of nascent proteins is impaired, a quality control system called UPR is activated [12], [13], [14]. Initially, UPR is an adaptive response in which the cells attempt to overcome the accumulation of misfolded proteins through augmenting protein folding capacity. However, when ER stress is excessive and prolonged, cells undergo apoptotic cell death. Thus, ER stress response has a conditional ability to protect the cells or activate cell death program. In the hearts, ER stress response has been shown to be activated in several pathological models including myocardial infarction, ischemia/reperfusion and pressure overload-induced hypertrophy [20], [21], [13], [22]. Pressure overload by transverse aortic constriction has been shown to induce prolonged ER stress during the transition from cardiac hypertrophy to heart failure [20]. AMP-activated protein kinase protects cardiac myocytes from hypoxic injury by attenuating ER stress [23]. Aberrant ER quality control in transgenic mice with mutant KDEL receptor, or chronic myocardial inflammation induced by chemoattractant protein-1 transgene activates ER stress response and causes heart failure [24], [25]. These results indicated that ER stress response could be deleterious in the hearts. In contrast, overexpression of ER stress gene GRP94 protects cardiac myocytes from oxidative injury [26], and inducible transgene of activated form of ATF6 in the hearts protects the hearts from ischemia/reperfusion damage [27], suggesting the protective role of ER stress response in the hearts. Thus, the outcome of ER stress response is also context dependent in the heart. In our model of hypertensive heart disease, ER stress response assessed by the expression of GRP78 and CHOP was activated in the phase of transition from hypertrophy to heart failure (Fig. 2C). While GRP78 expression reached a peak at 8 weeks of diet and then declined, CHOP expression remained upregulated until 12 weeks. Those expression patterns might be compatible with those reported in transverse aortic constriction-induced heart failure model, as a peak expression of GRP78 is observed at 1 week after transverse aortic constriction, while CHOP is upregulated even at 4 weeks [20]. Since induction of ER chaperone GRP78 represents a major survival arm of ER stress response, and indeed, overexpression of ER chaperone is protective for cardiac myocytes from ischemia and calcium overload injuries [26], these observations could be in good accordance with the general view that the UPR has a protective role during initial phase of ER stress, while proapoptotic pathways are activated upon continued ER stress [12], [13], [14]. Thus, ER stress response in our model might have the deleterious effect in the transition to heart failure, while it is still possible that this ER stress response was a part of counteracting efforts against developing heart failure.

Consistent with the contradictory roles of ER stress response in cell survival and death, ER stress has been demonstrated to activate both prosurvival and apoptotic signaling pathways [13], [14]. In cultured cardiac myocytes, treatment of cardiac myocytes with ER stress inducers resulted in reduced viability and increased apoptotic cell death [20]. In this setting, PARM-1 expression was markedly increased (Fig. 3B), and silencing PARM-1 expression significantly augmented ER stress-induced cell death (Fig. 4B), indicating PARM-1 is a part of cell survival pathways in ER stress response. Given that PARM-1 expression was induced in the phase of transition to heart failure in hypertensive heart disease model of Dahl salt-sensitive rats (Fig. 2B), the increased expression of PARM-1 in this model could play a role to counteract against development of heart failure through inhibiting apoptosis of cardiac myocytes, although further studies are needed to determine the role of PARM-1 induction in the development of heart failure in vivo.

ER stress is sensed by three distinct ER sensory proteins, PERK, ATF6 and IRE-1, which are kept inactivated by binding with ER-resident chaperones such as GRP78 and GRP94. Upon accumulation of misfolded proteins in ER, the chaperones are occupied by misfolded proteins, which results in the release and activation of the ER stress sensors, and subsequent activation of downstream signaling pathways [12], [13], [14]. The downstream signaling effectors include prosurvival and proapoptotic pathways, and it is anticipated that the balance between prosurvival and proapoptotic pathways determines the ultimate outcome of ER stress response, while the precise molecular mechanisms involved in cell fate determination remain to be delineated. In this study, silencing of PARM-1 did not alter the expression of GRP78, suggesting the expression of PARM-1 was not involved in the regulation of ER stress itself, or expression of ER chaperones (Fig. 5A). Downregulation of PARM-1 expression by siRNA markedly attenuated the expression of PERK and ATF6 without affecting IRE1 induction and XBP-1 splicing. Interestingly, these effects of PARM-1 silencing on PERK and ATF6 expression were only observed upon stimulation with thapsigargin or tunicamycin (Fig. 5). These results suggested that PARM-1 plays a crucial role in maintaining PERK and ATF6 expression in the setting of ER stress conditions. Since overall PERK signaling is protective against cell death in most circumstances [13], [14], [28], and preactivation of ATF6 protects the heart against ischemia/reperfusion insult [27], maintenance of PERK and ATF6 expression by PARM-1 is critical for cardiac myocytes to cope with ER stresses. The CHOP, JNK and Caspases are distal effectors of ER stress response that have been implicated in mediating apoptotic signals. CHOP induction was markedly augmented by PARM-1 silencing, while activation of JNK and Caspase-12 was unaffected (Fig. 5B,C), indicating that PARM-1 was also mediating prosurvival effect by suppressing CHOP-mediated apoptotic pathways in cardiac myocytes. Expression of CHOP, but not activation of JNK and Caspase-12 has been shown to be induced by transverse aortic constriction in murine hearts [20]. In cultured cardiac myocytes, proteasome inhibitors are shown to activate ER stress response and apoptosis, in which silencing CHOP, but not inhibition of JNK or Caspase-12 rescues cardiac myocytes from apoptosis [29]. Thus, CHOP expression might be one of important mechanisms inducing cardiac myocyte apoptosis in response to ER stress, and PARM-1 has an inhibitory role in this pathway. As CHOP is known to be regulated downstream of PERK and ATF-6 [30], [13], [28], and PARM-1 is involved in the maintenance of PERK and ATF-6 without affecting IRE-1 induction and XBP-1 splicing, it looks likely that PARM-1 is involved in a specific set of signal transduction pathways in ER stress response, while further studies are clearly needed to delineate the molecular mechanisms by which PARM-1 regulates ER stress response pathways.

An intriguing finding of this study was PARM-1 expression and induction of PARM-1 by ER stress was specific for cardiac myocytes (Fig. 3B). Recently, several ER membrane molecules specific for certain cell types have been identified. Those include CREB4, CREB-H, Luman and OASIS, and are involved in ER stress response [31], [32], [33], [34]. For example, CREB-H is expressed exclusively in the liver [35], and activated by ER stress [34]. However, ER stress-induced activation of CREB-H leads to induction of acute phase response genes such as C-reactive protein and serum amyloid P-component rather than canonical unfolded protein responses [34]. Thus, ER stress response could have specialized roles in specialized cells. Although there is a striking difference between those molecules and PARM-1, as those belong to basic-leucine zipper transcription factor, and PARM-1 does not have any known domain for transcription factors, PARM-1 could mediate ER stress response specific for cardiac myocytes.

In this study, we identified PARM-1 as an ER protein specifically expressed in cardiac myocytes, and found that PARM-1 expression was induced in hypertensive heart disease model, and by ER stress in cardiac myocytes. Further, it was also shown that PARM-1 had a protective role against ER stress-induced apoptotic response in cardiac myocytes. As elucidation of cardiac specific ER stress response could have therapeutic impacts on many heart diseases, identification of molecular mechanisms regulated by PARM-1 will be an important issue to understand cardiac specific ER stress responses.

## Materials and Methods

### Cardiac myocytes culture

Neonatal rat cardiac myocytes and non-myocytes were prepared from 1-day-old Wistar rat hearts as described previously [36], [37]. Neonatal rat ventricles were enzymatically digested, and cardiac myocytes were purified over a discontinuous Percoll gradient. Cardiac myocytes were cultured in DMEM/F-12 medium supplemented with 5% fetal bovine serum and 100 µmol/l 5-bromo-2-deoxyuridine (BrdU) for 16-24 hours, and culture medium was then changed to serum-deprived medium containing insulin, transferrin, selenium, bovine serum albumin and BrdU. Non-myocytes in the upper layer were plated onto non-coated culture dishes, and the attached cells were cultured and passaged. Non-myocytes at 2nd passage were used for the experiments. Each experiment was performed under serum free condition at least 24 hrs after serum deprivation.

### Signal sequence trap

Signal sequence trap by retrovirus-mediated expression screening (SST-REX) was performed as previously described [6], [9]. Briefly, a library was constructed in the retrovirus vector pMX-SST employing cDNA derived from poly(A)^+^ RNA isolated from neonatal rat cardiac myocytes. The interleukin-3 (IL-3)-dependent pro-B cell line Ba/F3 [6], [9] was infected with retrovirus, followed by seeding onto 96-multiwell plates in the absence of IL-3. Genomic DNA extracted from IL-3-independent Ba/F3 clones were subjected to PCR to recover the integrated cDNAs using primers specific for the cloning vector. After electrophoresis of the PCR products, DNA was recovered and subjected to sequencing.

### Animals and treatments

Male Dahl salt-sensitive rats (6 weeks old) were purchased from Shimizu laboratory supplies. From 6 weeks onwards, these rats were fed a high-sodium diet (containing 8% NaCl) or a low-sodium diet (containing 0.3% NaCl) [10], [11]. After 2, 4, 8 and 12 weeks, blood pressure was measured by the tail-cuff method. All procedures using animals performed in this study were approved by the Institutional Animal Care and Use Committee of Kyoto Prefectural University of Medicine.

### Reverse transcriptase (RT)-PCR

Total RNA was extracted from rat tissues using TRIzol (Invitrogen) and from neonatal rat cardiac myocytes using the RNeasy mini kit (Qiagen), and then cDNA was synthesized by the High Capacity cDNA Reverse Transcription Kit (Applied Biosystems). Synthesized cDNA was analyzed by quantitative kinetic real-time PCR using the ABI Prism 7700 Sequence Detector system (Applied Biosystems) with SYBR Premix Ex Taq (Takara) [38], [39]. Rat glyceraldehyde-3-phosphate dehydrogenase (GAPDH) was used for normalization, and the comparative threshold (C_T_) method was used to assess the relative abundance of the targets. For XBP-1 mRNA splicing, the primers flanking the intron splicing site by IRE-1 was used for PCR, and the products were analyzed by agarose gel electrophoresis with visualization using ethidium bromide [40].

### Immunostaining

Cells were stained with anti-flag M2 monoclonal antibody (Sigma) or goat polyclonal antibody against GRP78 (Santa Cruz), followed by Alexa Fluor 488- or Alexa Fluor 555-conjugated secondary antibody (Invitrogen). Mitochondria were stained with MitoTracker Green FM (Invitrogen) and nuclei were visualized using DAPI. Images were captured with a BZ-8000 microscope (Keyence).

### Immunoblot analysis

Cells lysates normalized by protein concentration were subjected to 10% or 15% SDS-polyacrylamide gel electrophoresis, and transferred to polyvinylidene difluoride membranes (Millipore) [38], [39]. Blots were immunoblotted with the primary antibody against GRP78, CHOP (Santa Cruz), phospho-PERK, PERK, IRE-1, phospho-JNK, JNK (Cell Signaling), Caspase-12 (Sigma) or ATF6 (AnaSpec), and horseradish peroxidase-labeled donkey secondary antibody, followed by enhanced chemiluminescence (GE Healthcare). Densitomeric analysis was performed using ImageJ software.

### Small interfering RNA–mediated silencing

PARM-1 stealth siRNA duplexes were purchased from Invitrogen. Target sequences of siRNA are: siRNA-1; 5′-GAACACAGTCTCGGCAGTCCTGAAA-3′, siRNA-2; 5′-TCCGCTTCCGTTACCTCTAACCACA-3′, siRNA-3; 5′-GCGGCATATCTGAAGATCAGGCATT-3′. Cells were transfected with 30 nmol/L siRNA duplex using Lipofectamine RNAiMAX reagent (Invitrogen) according to the manufacture's instruction [41]. Stealth RNAi negative control (Invitrogen) was used as a control.

### Cell viability and TUNEL assay

Cell viability was assessed using WST-8 (2-(2-methoxy-4-nitrophenyl)-3-(4-nitrophenyl)-5-(2,4-disulfophenyl)-2H-tetrazolium, monosodium salt) (Kishida Kagaku) according to the manufacturer's instruction. Apoptotic cells were detected by *in situ* terminal deoxynucleotidyl transferase-mediated biotinylated UTP nick end labeling (TUNEL) assay using ApopTag Red In Situ Apoptosis Detection Kit (Chemicon) as previously described [39]. Cells were nuclear stained with DAPI, and the TUNEL positive and total nuclei were counted under the fluorescent microscope (IX71, Olympus Corporation) in 5 view fields per well.

### Statistical analysis

All experiments were performed at least three times in duplicates. Data were expressed as means ± standard errors and analyzed by unpaired Student's *t*-test for comparisons between two groups, or one-way ANOVA with post hoc analysis for multiple comparisons. A value of p<0.05 was considered statistically significant.
